# Association between red cell distribution width and 30-day mortality in patients with sepsis-associated liver injury: a retrospective cohort study

**DOI:** 10.3389/fmed.2024.1510997

**Published:** 2024-12-18

**Authors:** Ting Ao, Yingxiu Huang, Peng Zhen, Ming Hu

**Affiliations:** Department of Infectious Diseases, Beijing Luhe Hospital, Capital Medical University, Beijing, China

**Keywords:** sepsis-associated liver injury, red cell distribution width, 30-day mortality, critical illness, sepsis

## Abstract

**Background:**

Sepsis-associated liver injury (SALI) is a critical component of sepsis-induced multiple organ dysfunction with high mortality. Identifying biomarkers for risk stratification is essential. Red cell distribution width (RDW), indicating variation in red blood cell volume, has been linked to adverse outcomes in various diseases. This study aimed to evaluate the association between RDW and 30-day mortality in SALI patients.

**Methods:**

A retrospective cohort study was conducted using data from the Medical Information Mart for Intensive Care-IV database. Patients admitted to the intensive care unit (ICU) with SALI were included. RDW was recorded within the initial 24 h. The primary outcome was 30-day mortality. A multivariable Cox regression analysis was performed to examine the relationship between RDW and mortality.

**Results:**

Among 529 SALI patients (mean age 68.7 years, 61.8% male), 46.1% had RDW > 15.5%. The 30-day mortality rate was 35.5%. RDW was significantly higher in non-survivors compared to survivors (17.2 ± 3.0 vs. 15.4 ± 2.3, *P* < 0.001). Cox regression identified RDW as an independent risk factor for 30-day mortality (HR 1.14, 95% CI 1.09 to 1.19, *P* < 0.001). Subgroup analyses demonstrated that the findings were consistent across the various groups.

**Conclusion:**

Elevated RDW is independently associated with higher 30-day mortality in patients with SALI, suggesting its potential role in risk stratification and clinical management.

## 1 Introduction

Sepsis, characterized by life-threatening organ dysfunction due to a dysregulated host response to infection, is a leading cause of global health burden (1). A global epidemiological study reported 48.9 million new cases of sepsis and 11.0 million sepsis-related deaths in 2017, accounting for 19.7% of global mortality (2). The liver plays a key role in maintaining metabolic balance and immune regulation (3, 4), particularly in the context of sepsis, where it is vital for orchestrating immune responses (5). Sepsis-associated liver injury (SALI) refers to liver dysfunction or injury induced by sepsis. Various factors, including pathogens, shock, excessive inflammatory responses, persistent microcirculatory failure, and oxidative stress, can lead to SALI (6). There are two main manifestations of SALI: ischemic hypoxic liver injury and sepsis-related cholestasis (7). There are no unified diagnostic criteria for SALI. The Surviving Sepsis Campaign Guidelines recommend using a total bilirubin > 2 mg/dL and an international normalized ratio (INR) > 1.5 as diagnostic criteria (8, 9). Mortality rates for SALI have been reported to be alarmingly high, ranging from 29.3 to 68% (10, 11). Given the high risk of mortality, early identification of SALI patients at risk of poor outcomes is essential for optimizing diagnostic and therapeutic strategies, ultimately improving patient prognosis.

Red cell distribution width (RDW) is derived from the standard deviation of erythrocyte volume based on the mean corpuscular volume. It serves as an indicator of anisocytosis, reflecting the variation in red blood cell size (12). Oxidative stress and inflammation are believed to shorten the lifespan of red blood cells and inhibit their maturation, leading to the premature release of immature erythrocytes into the bloodstream, thereby elevating RDW (13). These processes—oxidative stress and inflammation—are also key elements in the pathophysiology of sepsis. As a result, RDW may prove useful in identifying critically ill patients who require more intensive treatment. RDW has been recognized as an important prognostic marker in various acute conditions, such as heart failure (14), pancreatitis (15), kidney injury (16), stroke (17), and sepsis (18). Elevated RDW is consistently associated with worse clinical outcomes. The objective of this study is to assess the relationship between RDW and 30-day mortality in patients with SALI, while also investigating its potential utility as a prognostic indicator.

## 2 Material and methods

### 2.1 Data sources and setting

The study population was obtained from the MIMIC-IV database. This database is an electronic health record repository that includes extensive, high-quality, deidentified data on intensive care unit (ICU) patients from the Beth Israel Deaconess Medical Center (BIDMC) in Boston, MA, USA, covering the years between 2008 and 2019. Access to this dataset was granted under certification number 58844105. The Institutional Review Board at BIDMC approved a waiver of informed consent and permitted the use of this research resource. This cohort study followed the guidelines established by the Strengthening the Reporting of Observational Studies in Epidemiology (STROBE) statement (19).

### 2.2 Study population

Our study encompassed all eligible ICU patients from the MIMIC-IV database. We specifically targeted adults diagnosed with sepsis-3, characterized by an increase of at least 2 points in the Sequential Organ Failure Assessment (SOFA) score as a result of a dysregulated host response to infection (1). According to the Surviving Sepsis Campaign guidelines, the criteria for diagnosing sepsis-associated liver injury (SALI) include a total bilirubin level exceeding 2 mg/dL and an INR greater than 1.5 in patients with sepsis (10). These criteria are widely recognized in the literature. The study was restricted to patients with RDW measurements obtained within the first 24 h of ICU admission. The exclusion criteria included the following: (1) individuals younger than 18 years; (2) multiple hospital admissions, excluding their initial ICU admission; (3) absence of RDW data within 24 h of ICU admission or missing information on other variables.

### 2.3 Data extraction

Data pertinent to this study were obtained from the MIMIC-IV database through Structured Query Language (SQL) queries and were subsequently stored in PostgreSQL. At the time of sepsis diagnosis, initial records were systematically collected, including demographic details such as age, sex, and race; vital signs like heart rate and mean blood pressure (MBP); and laboratory findings that encompassed RDW, white blood cell count (WBC), platelet count, hemoglobin, hematocrit, glucose, blood urea nitrogen (BUN), creatinine, and alanine aminotransferase (ALT). Additionally, comorbidities were noted, including congestive heart failure, chronic respiratory disease, diabetes, renal disease, and malignant cancer, along with disease severity metrics such as SOFA score, Charlson comorbidity index, and simplified acute physiology score (SAPS II). The SOFA score evaluates organ dysfunction across six systems, each scored from 0 to 4, with higher scores indicating worse dysfunction (1) (Supplementary Table 1). The SAPS II score is calculated within 24 h of ICU admission, using 12 physiological variables (e.g., heart rate, blood pressure, oxygenation, serum potassium) and patient factors (age, chronic conditions) (20) (Supplementary Table 2). Each variable is assigned a predefined score, with higher scores indicating a higher mortality risk. The Charlson comorbidity index assesses 17 conditions, including cardiovascular disease, diabetes, chronic kidney disease, liver disease, and cancer (21) (Supplementary Table 3). Higher scores indicate a greater comorbidity burden and higher mortality risk. Interventions, including antibiotic therapy and vasoactive agents administered on day one, were also noted. Comorbidity information was obtained using the International Classification of Diseases (ICD) coding systems.

### 2.4 Grouping

Since none of the patients had an RDW below the normal range of 10.5 to 15.5%, they were classified into two groups: the normal RDW group (nRDW), comprising individuals with RDW ≤ 15.5%, and the high RDW group (hRDW), which included those with RDW > 15.5%. In addition, RDW was categorized into quartiles (Q1-Q4) for sensitivity analysis.

### 2.5 Outcome

The primary outcome was 30-day mortality. The secondary outcomes included in-hospital mortality, 90-day mortality, ventilator-free days until 28 days, vasopressor-free days until 28 days, and ICU-free days until 28 days.

### 2.6 Statistical analysis

Due to the retrospective design of this study, no predefined statistical analysis plan or power calculation was implemented; the sample size relied on available database data. Categorical variables are presented as frequencies and percentages, whereas continuous variables are expressed as means with standard deviations (SD) or medians with interquartile ranges (IQR). Continuous variables were assessed based on their distribution, utilizing either the Student’s *t*-test or the Wilcoxon rank-sum test. Categorical variables were evaluated using either Pearson’s chi-squared test or Fisher’s exact test.

Variable selection was guided by clinical relevance and existing literature. Variables with *p*-values < 0.1 in univariate analysis were included in the multivariate analysis (Supplementary Table 4). The effect of RDW on 30-day mortality was assessed using multivariable Cox regression models, yielding hazard ratios (HR) and 95% confidence intervals (CI) after adjusting for relevant covariates. Subgroup analyses were performed to explore interactions with covariates such as age, race, and comorbidities. Kaplan–Meier survival curves evaluated 30-day mortality across RDW categories, with significance determined by the log-rank test. Receiver operating characteristic (ROC) curves were generated to assess RDW’s predictive performance for 30-day mortality, calculated as the area under the curve (AUC). In addition, sensitivity analyses were performed by converting the types of variables and excluding special populations.

All analyses utilized R Statistical Software (Version 4.2.2, The R Foundation)^1^ and Free Statistics analysis platform (Version 2.0, Beijing, China). A *p*-value < 0.05 in a two-sided test was deemed statistically significant.

## 3 Results

### 3.1 Study cohort and patients’ characteristics

**FIGURE 1:**
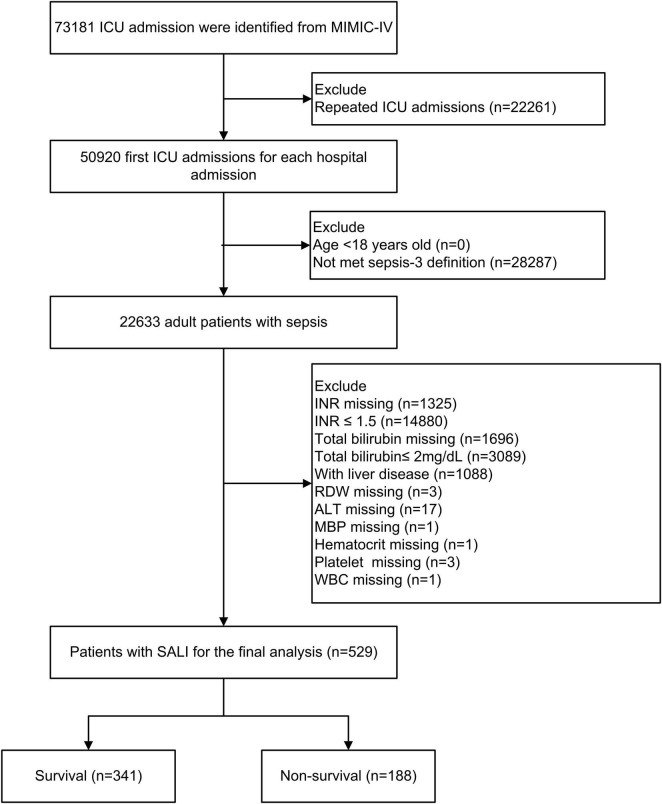
Flow chart of patient selection. ICU, intensive care unit; MIMIC-IV, Medical Information Mart in Intensive Care-IV; SALI, sepsis-associated liver injury; INR, international normalized ratio, RDW, red blood cell distribution width; MBP, mean blood pressure; WBC, white blood cell; ALT, alanine aminotransferase.

This analysis included a total of 529 patients diagnosed with SALI. The patient selection and data screening processes are illustrated in Figure 1. The baseline characteristics of patients, stratified by survival status, are presented in Table 1. The overall mean age at ICU admission was 68.7 years, and 61.8% of the patients were male. Patients in the non-survival group demonstrated higher scores on the Charlson comorbidity index, SOFA score, and SAPS II. Specifically, the RDW was significantly higher in the non-survival group compared to the survival group (17.2 vs. 15.4%, *P* < 0.001). Furthermore, the proportion of patients receiving vasopressin in the non-survival group was significantly greater than that in the survival group (75.5 vs. 54%, *P* < 0.001).

**TABLE 1:** Baseline characteristics of participants.

Patient characteristic	Total (*n* = 529)	Survival (*n* = 341)	Non-survival (*n* = 188)	*P*
Sex, *n* (%)				0.479
Female	202 (38.2)	134 (39.3)	68 (36.2)	
Male	327 (61.8)	207 (60.7)	120 (63.8)	
Age (years), Mean ± SD	68.7 ± 16.7	67.6 ± 17.5	70.8 ± 14.9	0.031
Race, *n* (%)				0.090
White	343 (64.8)	230 (67.4)	113 (60.1)	
Other	186 (35.2)	111 (32.6)	75 (39.9)	
**Vital signs**				
Heart rate (bpm), mean ± SD	93.9 ± 18.2	91.3 ± 17.0	98.4 ± 19.4	< 0.001
MBP (mmHg), mean ± SD	74.6 ± 9.1	75.9 ± 8.7	72.4 ± 9.3	< 0.001
**Comorbidity disease, n (%)**				
Congestive heart failure	216 (40.8)	132 (38.7)	84 (44.7)	0.181
Chronic pulmonary disease	114 (21.6)	75 (22)	39 (20.7)	0.738
Diabetes	152 (28.7)	100 (29.3)	52 (27.7)	0.685
Renal disease	120 (22.7)	71 (20.8)	49 (26.1)	0.168
Malignant cancer	115 (21.7)	54 (15.8)	61 (32.4)	< 0.001
**Score system**				
Charlson comorbidity index, median (IQR)	6.0 (4.0, 8.0)	5.0 (4.0, 7.0)	7.0 (5.0, 9.0)	< 0.001
SOFA score, median (IQR)	4.0 (3.0, 6.0)	4.0 (3.0, 6.0)	5.0 (4.0, 7.0)	0.011
SAPS II, mean ± SD	50.5 ± 17.6	44.2 ± 15.0	61.9 ± 16.3	< 0.001
**Laboratory parameters**				
Hemoglobin (g/dL), mean ± SD	10.6 ± 2.5	10.7 ± 2.5	10.3 ± 2.7	0.064
Platelets (K/uL), median (IQR)	145.0 (89.0, 204.0)	144.0 (96.0, 200.0)	146.5 (72.8, 209.0)	0.485
WBC (K/uL), median (IQR)	13.4 (7.9, 20.0)	13.4 (8.4, 20.1)	13.3 (7.2, 19.9)	0.299
Hematocrit (%), mean ± SD	32.3 ± 7.7	32.5 ± 7.5	31.8 ± 8.2	0.325
Creatinine (mg/dL), median (IQR)	1.4 (0.9, 2.2)	1.2 (0.9, 1.7)	1.8 (1.2, 2.8)	< 0.001
BUN (mg/dL), median (IQR)	27.0 (17.0, 46.0)	23.0 (15.0, 34.0)	40.5 (22.8, 61.2)	< 0.001
Glucose (mg/dL), median (IQR)	128.0 (101.0, 162.0)	127.0 (103.0, 158.0)	130.0 (96.0, 174.0)	0.856
ALT (IU/L), median (IQR)	93.0 (33.0, 244.0)	100.0 (35.0, 228.0)	84.5 (31.0, 324.2)	0.761
RDW (%), mean ± SD	16.0 ± 2.7	15.4 ± 2.3	17.2 ± 3.0	< 0.001
RDW				< 0.001
≤ 15.5	285 (53.9)	218 (63.9)	67 (35.6)	
> 15.5	244 (46.1)	123 (36.1)	121 (64.4)	
**Interventions, n (%)**				
Antibiotic (day 1), *n* (%)	496 (93.8)	320 (93.8)	176 (93.6)	0.919
Vasoactive agent (day 1), *n* (%)	326 (61.6)	184 (54)	142 (75.5)	< 0.001

bpm, beats per minute; MBP, mean blood pressure; SD, standard deviation; IQR, interquartile range; SOFA, Sequential Organ Failure Assessment; SAPS II, simplified acute physiology score; BUN, blood urea nitrogen; WBC, white blood cell; ALT, alanine aminotransferase; RDW, red blood cell distribution width.

### 3.2 Association between RDW and 30-day mortality

The overall prevalence of 30-day mortality among the cohort was found to be 35.5%. When RDW was considered as a continuous variable, the univariable Cox regression analysis revealed a significant association with 30-day mortality (crude HR 1.17; 95% CI, 1.13 to 1.23; *P* < 0.001). Following multivariable adjustment for factors including age, race, heart rate, MBP, creatinine, BUN, ALT, Charlson comorbidity index, SOFA, SAPS II, malignant cancer, and vasoactive agent, this association remained significant (adjusted HR 1.14; 95% CI, 1.09 to 1.19; *P* < 0.001) (Table 2). To further investigate this relationship, we utilized a restricted cubic spline analysis. The result indicated that, after accounting for the previously mentioned covariates, the relationship between RDW and 30-day mortality was linear (*P* for non-linearity = 0.340) (Supplementary Figure 1).

**TABLE 2:** Relationship between red blood cell distribution width and 30-day morality in different models.

Item	N. total	N. event (%)	Model 1		Model 2		Model 3	
			**HR (95% CI)**	***p*-value**	**HR (95% CI)**	***p*-value**	**HR (95% CI)**	***p*-value**
RDW	529	188 (35.5)	1.17 (1.13∼1.23)	< 0.001	1.18 (1.13∼1.23)	< 0.001	1.14 (1.09∼1.19)	< 0.001
**RDW-group**								
≤ 15.5	285	67 (23.5)	1 (Ref)		1 (Ref)		1 (Ref)	
> 15.5	244	121 (49.6)	2.41 (1.79∼3.25)	< 0.001	2.35 (1.75∼3.18)	< 0.001	1.62 (1.17∼2.23)	0.004
**RDW, quartiles**								
Q1	128	19 (14.8)	1 (Ref)		1 (Ref)		1 (Ref)	
Q2	126	34 (27.0)	1.95 (1.11∼3.42)	0.02	1.88 (1.07∼3.3)	0.028	1.54 (0.87∼2.71)	0.137
Q3	140	55 (39.3)	2.98 (1.77∼5.03)	< 0.001	2.83 (1.68∼4.78)	< 0.001	1.70 (0.99∼2.92)	0.054
Q4	135	80 (59.3)	5.22 (3.16∼8.61)	< 0.001	5.06 (3.07∼8.36)	< 0.001	3.71 (2.19∼6.28)	< 0.001
Trend test				< 0.001		< 0.001		< 0.001

Model 1: unadjusted. Model 2: adjust for age, race. Model 3: adjust for model 2+heart rate, MBP, creatinine, BUN, ALT, Charlson comorbidity index, SOFA, SAPS II, malignant cancer, vasoactive agent (day 1). HR, hazard ratio; CI, confidence interval; RDW, red blood cell distribution width; MBP, mean blood pressure; SOFA, Sequential Organ Failure Assessment; SAPS II, simplified acute physiology score; BUN, blood urea nitrogen; ALT, alanine aminotransferase.

**FIGURE 2:**
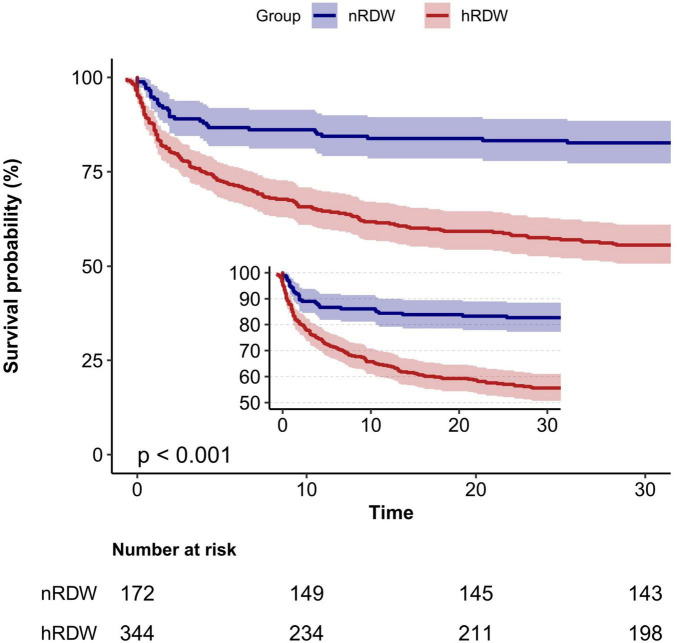
Kaplan–Meier curve for 30-day mortality according to red blood cell distribution width (RDW). nRDW, the normal RDW group (RDW ≤ 15.5%); hRDW, the high RDW group (RDW > 15.5%).

When RDW was treated as a categorical variable with a cutoff of 15.5%, the hRDW group exhibited a heightened risk of 30-day mortality compared with the nRDW group (adjusted HR 1.62; 95% CI, 1.17 to 2.23; *P* = 0.004) (Table 2). Kaplan–Meier survival curve estimates further indicated a significantly elevated risk of 30-day mortality in patients with high RDW (log-rank: *P* < 0.001; Figure 2). The association persisted when RDW was categorized into quartiles. Compared to individuals in Q1, those in Q4 faced a 2.71-fold increased risk of 30-day mortality (adjusted HR, 3.71; 95% CI, 2.19 to 6.28; *P* < 0.001; Table 2, Model 3).

### 3.3 Subgroup analyses

**FIGURE 3:**
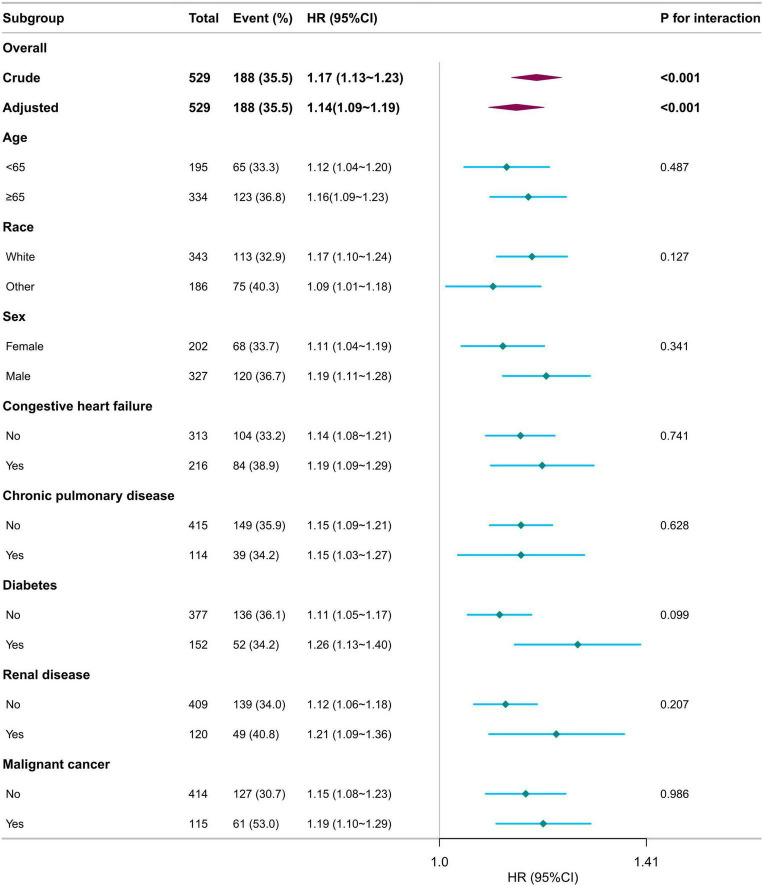
Subgroup analyses for the association of red blood cell distribution width with 30-day mortality in patients with sepsis-associated liver injury. Multivariate Cox proportional hazards models were adjusted for age, race, heart rate, mean blood pressure, creatinine, blood urea nitrogen, alanine aminotransferase, Charlson comorbidity index, sequential organ failure assessment score, simplified acute physiology score II, malignant cancer, vasoactive agent (day 1).

Subgroup analyses were conducted to assess the influence of age, sex, race, congestive heart failure, diabetes, chronic pulmonary disease, renal disease, and malignant cancer on the relationship between RDW and 30-day mortality. No statistically significant interactions were identified within any subgroup (*p* > 0.05, Figure 3). These results indicate that the effect of RDW on 30-day mortality remains consistent across different age, race, sex, and comorbidity groups.

### 3.4 ROC curve analysis

**FIGURE 4:**
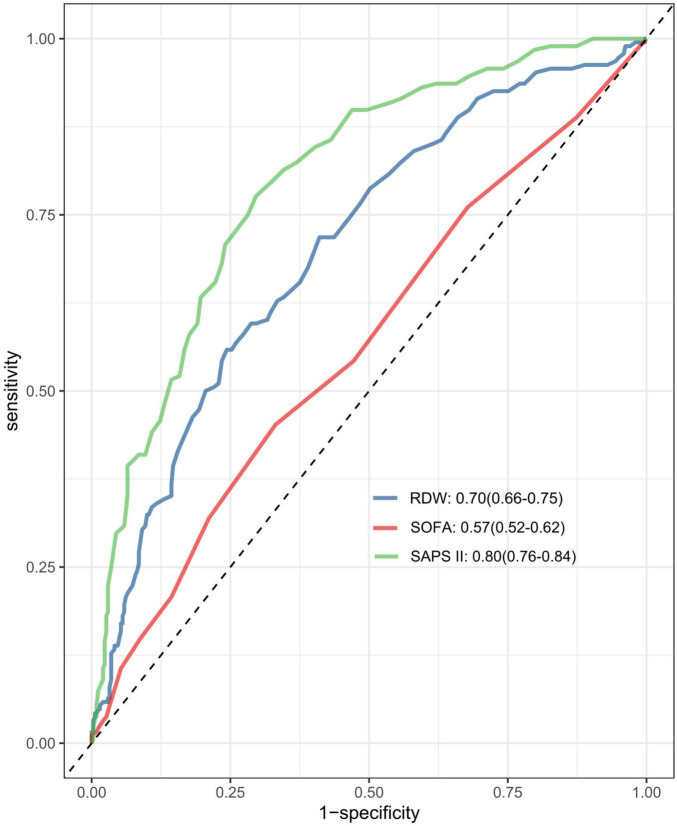
The area under the receiver operating characteristic curve (AUC-ROC) for the models including RDW, SAPS II, and SOFA score. RDW, red blood cell distribution width SOFA, Sequential Organ Failure Assessment; SAPS II, simplified acute physiology score.

ROC analysis revealed that RDW possesses moderate clinical predictive value for 30-day mortality in patients with SALI, achieving an AUC of 0.70 (95% CI, 0.66 to 0.75). We subsequently compared the discriminatory ability for 30-day in-hospital mortality among RDW, SAPS II, and SOFA using ROC curves (Figure 4). RDW exhibited stronger predictive discrimination for 30-day mortality in SALI patients compared to SOFA (AUC, 0.57; 95% CI, 0.52 to 0.62; *P* < 0.001), but was less effective than SAPS II (AUC, 0.80; 95% CI, 0.76 to 0.84; *P* = 0.002) (Supplementary Figures 2–4 and Supplementary Table 5).

### 3.5 Secondary outcomes

Increased RDW levels were associated with a higher risk of in-hospital mortality (adjusted HR, 1.13; 95% CI, 1.07 to 1.18; *P* < 0.001). Similar patterns were observed for 90-day mortality. Each 1% rise in RDW was linked to a reduction of 0.81 days in ICU-free days (95% CI, −1.12 to −0.51), 0.96 days in ventilator-free days (95% CI, −1.30 to −0.62), and 0.98 days in vasopressor-free days (95% CI, −1.32 to −0.65) over a 28-day period (Table 3).

**TABLE 3:** Secondary outcome analysis.

Secondary outcomes	N. total	N. event (%)	Crude model		Adjusted model	
			**HR/β (95% CI)**	***p*-value**	**HR/β (95% CI)**	***p*-value**
In-hospital mortality	529	166 (31.4)	1.15 (1.10∼1.21)	< 0.001	1.13 (1.07∼1.18)	< 0.001
90-day mortality	529	224 (42.3)	1.18 (1.13∼1.22)	< 0.001	1.14 (1.09∼1.19)	< 0.001
ICU-free days until 28 days	529		−1.26 (−1.61∼−0.91)	< 0.001	−0.81 (−1.12∼−0.51)	< 0.001
Vasopressor-free days until 28 days	529		−1.49 (−1.87∼−1.11)	< 0.001	−0.98 (−1.32∼−0.65)	< 0.001
Ventilator-free days until 28 days	529		−1.47 (−1.86∼−1.09)	< 0.001	−0.96 (−1.30∼−0.62)	< 0.001

Crude model: unadjusted. Adjusted model: adjusted for age, race, heart rate, MBP, creatinine, BUN, ALT, Charlson comorbidity index, SOFA, SAPS II, malignant cancer, vasoactive agent (day 1). HR, hazard ratio; CI, confidence interval; ICU, intensive care unit; MBP, mean blood pressure; SOFA, Sequential Organ Failure Assessment; SAPS II, simplified acute physiology score; BUN, blood urea nitrogen; ALT, alanine aminotransferase.

### 3.6 Sensitivity analysis

We converted non-normally distributed continuous variables, including BUN, creatinine, ALT, SOFA score, and Charlson comorbidity index, into categorical variables for sensitivity analysis. BUN, creatinine, and ALT were classified according to clinically relevant cut-offs. Normal values range from 6 to 20 mg/dL for BUN and 0.5 to 1.2 mg/dL for creatinine, and 0–40 IU/L for ALT. The SOFA score and Charlson comorbidity index were classified according to the median, which were 4 and 6, respectively. The multivariable Cox regression analysis showed that higher RDW was significantly associated with an increased risk of 30-day mortality (Supplementary Table 6).

In addition, we performed a multivariate COX regression analysis after excluding patients with liver malignancies, anemia, and myelodysplastic syndromes, and the results remained robust (Supplementary Tables 7, 8).

## 4 Discussion

In this retrospective cohort study that employed extensive public datasets, we discovered a notable correlation between RDW and 30-day mortality among patients with SALI. The multivariate Cox regression analysis, after controlling for potential confounders, consistently demonstrated an elevated risk of 30-day mortality among SALI patients with higher RDW levels. This association was consistently evident across various subgroup analyses. Additionally, the Kaplan–Meier survival curve analysis showed a greater 30-day mortality rate in the hRDW group. ROC analysis further indicated that RDW possesses moderate clinical predictive value for 30-day mortality in SALI patients. Our findings provide substantial evidence supporting the potential role of RDW as a prognostic biomarker for mortality prediction in this population.

A growing body of evidence has established a link between RDW and negative outcomes across various diseases. Numerous studies have identified RDW as an independent prognostic factor for sepsis (22, 23). Melchio et al. (14) found that RDW was a powerful marker of worse long-term outcomes in patients with acute heart failure, and its prognostic value is maintained beyond that provided by other well-established risk factors or biomarkers. O’Connell et al. (15) demonstrated that patients with RDW levels exceeding the normal upper limit faced a greater risk of inpatient mortality and an increased likelihood of requiring critical care support. In both retrospective and prospective analyses involving 258 patients with ARDS, those with RDW ≥ 14.45% exhibited significantly higher rates of acute kidney injury and 28-day mortality compared to those with RDW < 14.45% (16). Additionally, a separate study focused on identifying biomarkers for predicting in-hospital mortality in SALI patients, which involved 770 individuals, and identified a significant association between RDW and mortality (7). Our findings are consistent with these results, indicating that RDW is associated with 30-day mortality in patients with SALI and may function as a valuable prognostic marker.

Nevertheless, some researchers have raised doubts regarding the prognostic significance of RDW. Fontana et al. (24) carried out a study with 122 patients suffering from sepsis and reported no relationship between RDW and microcirculatory alterations, disease severity, the presence of shock, or survival outcomes. Another study observed that while RDW was linked to 30-day mortality in patients with severe sepsis, it did not serve as an independent predictor of mortality (25). These inconsistencies may stem from variations in study populations, the timing of measurements, and the criteria used to define abnormal RDW levels.

Various scoring systems, such as SOFA and SAPS II, are commonly used to predict outcomes in critically ill patients (26), but their effectiveness is often questioned. These scores involve multiple indicators, making them cumbersome, especially in resource-limited settings. In comparison, RDW is straightforward to acquire, quickly measurable, and offers relatively high accuracy, making it a convenient index for prognostic prediction. Our ROC analysis indicates that RDW provides a moderate prediction of 30-day mortality in SALI patients, demonstrating better predictive capability than the SOFA score while being less effective than the SAPS II score. Therefore, RDW may have a certain complement to existing biomarkers in predicting mortality. Further prospective studies are needed in the future to verify its role in clinical practice.

The exact mechanisms linking increased RDW to mortality in SALI patients remain unclear. Research has demonstrated a notable association between RDW and inflammatory markers (12). Proinflammatory cytokines may damage red blood cell membranes and inhibit maturation, resulting in the entry of larger, immature erythrocytes into circulation and subsequently raising RDW. Additionally, heightened oxidative stress could reduce erythrocyte lifespan, prompting the release of larger immature cells from the bone marrow (27). However, since this study is a retrospective observational analysis, it cannot elucidate the underlying mechanisms. More research is needed to investigate these processes and better understand the role of RDW in liver injury among septic patients.

This study presents several strengths. Firstly, it benefits from a relatively large sample size, with data sourced from the MIMIC-IV database, a comprehensive and high-quality real-world resource. Secondly, the robustness of the findings is supported by subgroup analyses. However, there are limitations to consider. Primarily, as a retrospective study, it is prone to selection bias. Secondly, due to the limitations of public databases, we were unable to exclude all patients with diseases that could affect our results, such as secondary sclerosing cholangitis in critically ill patients (SSC-CIP). However, we have tried to exclude patients with liver diseases such as chronic liver disease/cirrhosis as much as possible to ensure the accuracy and specificity of our findings. In addition, we further excluded people with liver malignancies, anemia, and myelodysplastic syndromes and conducted a sensitivity analysis to verify the reliability of our results. Thirdly, due to the nature of our retrospective study design and the limitations inherent in the dataset we utilized, we were unable to access detailed information regarding the specific methods used for laboratory tests. Additionally, we only included RDW data for patients upon ICU admission, preventing us from evaluating how changes in RDW post-admission might influence mortality, potentially affecting result accuracy. Furthermore, despite efforts to account for confounding variables, the retrospective design leaves open the possibility of unidentified confounders impacting our findings. Lastly, the study’s design does not permit conclusions about causality between RDW and mortality.

## 5 Conclusion

Elevated RDW levels are significantly associated with increased 30-day mortality in patients with SALI, indicating that RDW may serve as a reliable and effective prognostic indicator for sepsis. Nonetheless, further large-scale prospective multicenter studies are required to confirm these findings.

## Data Availability

The datasets presented in this study can be found in online repositories. The names of the repository/repositories and accession number(s) can be found below: This study used the MIMIC IV v2.2 dataset. This dataset can be found in the online repository at https://physionet.org/content/mimiciv/2.2/.

## Footnotes

1
http://www.r-project.org

## References

[1] Singer M Deutschman C Seymour C Shankar-Hari M Annane D Bauer M The third international consensus definitions for sepsis and septic shock (sepsis-3). *JAMA.* (2016) 315(8):801–10. 26903338 10.1001/jama.2016.0287 PMC4968574

[2] Rudd K Johnson S Agesa K Shackelford K Tsoi D Kievlan D Global, regional, and national sepsis incidence and mortality, 1990-2017: Analysis for the global burden of disease study. *Lancet Lond Engl.* (2020) 395(10219):200–11. 10.1016/S0140-6736(19)32989-7 PMC6970225 31954465

[3] Solhi R Lotfinia M Gramignoli R Najimi M Vosough M. Metabolic hallmarks of liver regeneration. *Trends Endocrinol Metab.* (2021) 32(9):731–45. 34304970 10.1016/j.tem.2021.06.002

[4] Kubes P Jenne C. Immune responses in the liver. *Annu Rev Immunol.* (2018) 36:247–77. 29328785 10.1146/annurev-immunol-051116-052415

[5] Zhang X Liu H Hashimoto K Yuan S Zhang J. The gut-liver axis in sepsis: Interaction mechanisms and therapeutic potential. *Crit Care Lond Engl.* (2022) 26(1):213. 10.1186/s13054-022-04090-1 PMC9277879 35831877

[6] Lelubre C Vincent J. Mechanisms and treatment of organ failure in sepsis. *Nat Rev Nephrol.* (2018) 14(7):417–27. 29691495 10.1038/s41581-018-0005-7

[7] Liu Y Sun R Jiang H Liang G Huang Z Qi L Development and validation of a predictive model for in-hospital mortality in patients with sepsis-associated liver injury. *Ann Transl Med.* (2022) 10(18):997. 10.21037/atm-22-4319 PMC9577780 36267798

[8] Wen C Zhang X Li Y Xiao W Hu Q Lei X An interpretable machine learning model for predicting 28-day mortality in patients with sepsis-associated liver injury. *PLoS One.* (2024) 19(5):e0303469. 10.1371/journal.pone.0303469 38768153 PMC11104601

[9] Dellinger R Levy M Rhodes A Annane D Gerlach H Opal S Surviving sepsis campaign: International guidelines for management of severe sepsis and septic shock: 2012. *Crit Care Med.* (2013) 41(2):580. 10.1097/CCM.0b013e31827e83af 23353941

[10] Yi X Jin D Huang S Xie Z Zheng M Zhou F Association between lactate-to-albumin ratio and 28-days all-cause mortality in patients with sepsis-associated liver injury: A retrospective cohort study. *BMC Infect Dis.* (2024) 24(1):65. 10.1186/s12879-024-08978-x 38195421 PMC10775525

[11] Zhang Z Tan X Shi H Zhang H Li J Liao X. Bibliometric study of sepsis-associated liver injury from 2000 to 2023. *World J Gastroenterol.* (2024) 30(30):3609–24. 10.3748/wjg.v30.i30.3609 39193568 PMC11346150

[12] Salvagno G Sanchis-Gomar F Picanza A Lippi G. Red blood cell distribution width: A simple parameter with multiple clinical applications. *Crit Rev Clin Lab Sci.* (2015) 52(2):86–105. 10.3109/10408363.2014.992064 25535770

[13] Bazick H Chang D Mahadevappa K Gibbons F Christopher K. Red cell distribution width and all-cause mortality in critically ill patients. *Crit Care Med.* (2011) 39(8):1913–21. 21532476 10.1097/CCM.0b013e31821b85c6 PMC4427349

[14] Melchio R Rinaldi G Testa E Giraudo A Serraino C Bracco C Red cell distribution width predicts mid-term prognosis in patients hospitalized with acute heart failure: The RDW in acute heart failure (RE-AHF) study. *Intern Emerg Med.* (2019) 14(2):239–47. 10.1007/s11739-018-1958-z 30276661

[15] O’Connell R Boland M O’Driscoll J Salih A Arumugasamy M Walsh T Red cell distribution width and neutrophil to lymphocyte ratio as predictors of outcomes in acute pancreatitis: A retrospective cohort study. *Int J Surg Lond Engl.* (2018) 55:124–7. 10.1016/j.ijsu.2018.05.028 29807170

[16] Cai N Jiang M Wu C He F. Red cell distribution width at admission predicts the frequency of acute kidney injury and 28-day mortality in patients with acute respiratory distress syndrome. *Shock Augusta Ga.* (2022) 57(3):370–7. 10.1097/SHK.0000000000001840 34606226 PMC8868185

[17] Xie K Liu L Liang Y Su C Li H Liu R Red cell distribution width: A novel predictive biomarker for stroke risk after transient ischaemic attack. *Ann Med.* (2022) 54(1):1167–77. 10.1080/07853890.2022.2059558 35471128 PMC9045760

[18] Dankl D Rezar R Mamandipoor B Zhou Z Wernly S Wernly B Red cell distribution width is independently associated with mortality in sepsis. *Med Princ Pract.* (2022) 31(2):187–94. 35093953 10.1159/000522261 PMC9209973

[19] von Elm E Altman D Egger M Pocock S Gøtzsche P Vandenbroucke J The strengthening the reporting of observational studies in epidemiology (STROBE) statement: Guidelines for reporting observational studies. *Lancet Lond Engl.* (2007) 370(9596):1453–7. 10.1016/S0140-6736(07)61602-X 18064739

[20] Le Gall J Lemeshow S Saulnier FA. new simplified acute physiology score (SAPS II) based on a european/north american multicenter study. *JAMA.* (1993) 270(24):2957–63. 8254858 10.1001/jama.270.24.2957

[21] Charlson M Pompei P Ales K MacKenzie CRA. new method of classifying prognostic comorbidity in longitudinal studies: development and validation. *J Chronic Dis.* (1987) 40(5):373–83. 3558716 10.1016/0021-9681(87)90171-8

[22] Jiang Y Jiang F Kong F An M Jin B Cao D Inflammatory anemia-associated parameters are related to 28-day mortality in patients with sepsis admitted to the ICU: A preliminary observational study. *Ann Intensive Care.* (2019) 9(1):67. 10.1186/s13613-019-0542-7 31183575 PMC6557959

[23] Kim Y Song J Kim E Choi H Jeong W Jung I A simple scoring system using the red blood cell distribution width, delta neutrophil index, and platelet count to predict mortality in patients with severe sepsis and septic shock. *J Intensive Care Med.* (2019) 34(2):133–9. 30021478 10.1177/0885066618787448

[24] Fontana V Spadaro S Bond O Cavicchi F Annoni F Donadello K No relationship between red blood cell distribution width and microcirculatory alterations in septic patients. *Clin Hemorheol Microcirc.* (2017) 66(2):131–41. 10.3233/CH-160154 28128746

[25] Jandial A Kumar S Bhalla A Sharma N Varma N Varma S. Elevated red cell distribution width as a prognostic marker in severe sepsis: A prospective observational study. *Indian J Crit Care Med.* (2017) 21(9):552–62. 28970653 10.4103/ijccm.IJCCM_208_17 PMC5613605

[26] Simpson S. New sepsis criteria: A change we should not make. *Chest.* (2016) 149(5):1117–8. 26927525 10.1016/j.chest.2016.02.653

[27] Karabulut B Arcagok B. New diagnostic possibilities for early onset neonatal sepsis: Red cell distribution width to platelet ratio. *Fetal Pediatr Pathol.* (2020) 39(4):297–306. 10.1080/15513815.2019.1661051 31510842

[28] Vincent JL Moreno R Takala J Willatts S De Mendonça A Bruining H The SOFA (Sepsis-related Organ Failure Assessment) score to describe organ dysfunction/failure. On behalf of the Working Group on Sepsis-Related Problems of the European Society of Intensive Care Medicine. *Intensive Care Med*. (1996) 22:707–10. 10.1007/BF01709751 8844239

