# Supplementary material for: Association between red cell distribution width and 30-day mortality in patients with sepsis-associated liver injury: a retrospective cohort study
Source: Front Med (Lausanne). 2024 Dec 18;11:1510997. doi: 10.3389/fmed.2024.1510997 (PMC11688371; doi:10.3389/fmed.2024.1510997)
Supplement: Supplementary file 1 [file Table_1.docx]

| system | score | | | | |
| --- | --- | --- | --- | --- | --- |
|  | 0 | 1 | 2 | 3 | 4 |
| respiration | | | | | |
| PaO_2_/FIO_2_, mmHg(kPa) | ≥400 (53.3) | <400 (53.3) | <300 (40) | <200 (26.7) with respiratory support | <100 (13.3) with respiratory support |
| Coagulation | | | | | |
| Platelets, ×10^3^/µL | ≥150 | <150 | <100 | <50 | <20 |
| Liver | | | | | |
| Bilirubin, mg/dL | <1.2 (20) | 1.2–1.9 (20–32) | 2.0–5.9 (33–101) | 6.0–11.9 (102–204) | >12.0 (204) |
| Cardiovascular | MAP ≥70mmHg | MAP <70mmHg | Dopamine <5 or dobutamine (any dose) | Dopamine 5.1-15 or epinephrine≤0.1 or norepinephrine ≤0.1 | Dopamine >15 or epinephrine >0.1 or norepinephrine >0.1 |
| Central nervous system | | | | | |
| Glasgow Coma Scale score | 15 | 13–14 | 10–12 | 6–9 | <6 |
| Renal | | | | | |
| Creatinine, mg/dL(µmol/L) | <1.2 (110) | 1.2–1.9 (110–170) | 2.0–3.4 (171–299) | 3.5–4.9 (300–440) | >5.0 (440) |
| Urine output, mL/d |  |  |  | <500 | <200 |

Supplementary Table 1 Sequential Organ Failure Assessment Score^a^

Note: ^a^ Data sourced from Singer et al. (2016) (1) and Vincent et al (1996) (28).
