# Supplementary material for: Association between red cell distribution width and 30-day mortality in patients with sepsis-associated liver injury: a retrospective cohort study
Source: Front Med (Lausanne). 2024 Dec 18;11:1510997. doi: 10.3389/fmed.2024.1510997 (PMC11688371; doi:10.3389/fmed.2024.1510997)
Supplement: Supplementary file 2 [file Table_2.docx]

Supplementary Table 2 simplified acute physiology score^a^

| Variable | Points | Range |
| --- | --- | --- |
| Age, years | 0 | <40 |
|  | 7 | 40-59 |
|  | 12 | 60-69 |
|  | 15 | 70-74 |
|  | 16 | 75-79 |
|  | 18 | ≥80 |
| Heart Rate, bpm | 0 | 70-119 |
|  | 2 | 40-69 |
|  | 4 | 120-159 |
|  | 7 | ≥160m |
|  | 11 | <40 |
| Systolic Blood Pressure，mmHg | 0 | 100-199 |
|  | 2 | ≥200 |
|  | 5 | 70-99 |
|  | 13 | <70 |
| Temperature,°C | 0 | <39 |
|  | 3 | ≥39 |
| only if ventilated or continuous pulmonary artery pressure PaO2/FiO2 Ratio, mmHg | 6 | ≥200 |
|  | 9 | 100-199 |
|  | 11 | <100 |
| Urinary Output (L/d) | 0 | ≥1 |
|  | 5 | 0.5-1 |
|  | 11 | <0.5 |
| Serum Urea Nitrogen (mg/dL) | 0 | <28 |
|  | 6 | 28-83 |
|  | 10 | ≥84 |
| WBC Count (x10^3/μL) | 0 | 1-19.9 |
|  | 3 | ≥20 |
|  | 12 | <1 |
| Serum Potassium (mEq/L) | 0 | 3-4.9 |
|  | 3 | <3 or ≥5 |
| Serum Sodium (mEq/L) | 0 | 125-144 |
|  | 1 | ≥145 |
|  | 5 | <125 |
| Serum Bicarbonate (mEq/L) | 0 | ≥20 |
|  | 3 | 15-19 |
|  | 5 | <15 |
| Bilirubin (mg/dL) | 0 | <4 |
|  | 4 | 4-5.9 |
|  | 9 | ≥6 |
| Glasgow Coma Scale | 0 | 14-15 |
|  | 5 | 11-13 |
|  | 7 | 9-10 |
|  | 13 | 6-8 |
|  | 26 | <6 |
| Hematologic Malignancy | 0 | No |
|  | 10 | Yes |
| Metastatic Cancer | 0 | No |
|  | 9 | Yes |
| AIDS | 0 | No |
|  | 17 | Yes |
| type of admission | 0 | Scheduled Surgical |
|  | 6 | Medical |
|  | 8 | Unscheduled Surgical |

Note: ^a^ Data sourced from Le et al. (1993) (20).
