# Supplementary material for: Association between red cell distribution width and 30-day mortality in patients with sepsis-associated liver injury: a retrospective cohort study
Source: Front Med (Lausanne). 2024 Dec 18;11:1510997. doi: 10.3389/fmed.2024.1510997 (PMC11688371; doi:10.3389/fmed.2024.1510997)
Supplement: Supplementary file 3 [file Table_3.docx]

Supplementary Table 3 Charlson comorbidity index^a^

| Conditions | Score |
| --- | --- |
| Age, year |  |
| ≤40 | 0 |
| 40-50 | 1 |
| 50-60 | 2 |
| 60-70 | 3 |
| >70 | 4 |
| Myocardial infarct | 1 |
| Congestive heart failure | 1 |
| Peripheral vascular disease | 1 |
| Cerebrovascular disease | 1 |
| Dementia | 1 |
| Chronic pulmonary disease | 1 |
| rheumatic disease | 1 |
| Peptic ulcer disease | 1 |
| Mild liver disease | 1 |
| Diabetes without complication | 1 |
| Diabetes with complication | 1 |
| paraplegia | 1 |
| renal disease | 1 |
| Malignant cancer | 1 |
| severe liver disease | 1 |
| Metastatic solid tumor | 1 |
| Acquired Immune Deficiency Syndrome | 1 |

Note: ^a^ Data sourced from Charlson et al. (1987) (21).
