# Supplementary material for: Association between red cell distribution width and 30-day mortality in patients with sepsis-associated liver injury: a retrospective cohort study
Source: Front Med (Lausanne). 2024 Dec 18;11:1510997. doi: 10.3389/fmed.2024.1510997 (PMC11688371; doi:10.3389/fmed.2024.1510997)
Supplement: Supplementary file 4 [file Table_4.docx]

Supplementary Table 4 Association of covariates and 30-day morality in patients with SALI.

| Item | HR (95%CI) | p-value |
| --- | --- | --- |
| Sex | 1.10 (0.81,1.48) | 0.541 |
| Age | 1.01 (1.00,1.02) | 0.033 |
| Race | 1.32 (0.99,1.77) | 0.063 |
| Heart rate | 1.02 (1.01,1.03) | < 0.001 |
| MBP | 0.96 (0.95,0.98) | < 0.001 |
| Congestive heart failure | 1.18 (0.89,1.57) | 0.257 |
| Chronic pulmonary disease | 0.93 (0.66,1.33) | 0.696 |
| Diabetes | 0.92 (0.67,1.27) | 0.628 |
| Renal disease | 1.25 (0.91,1.74) | 0.172 |
| Malignant cancer | 1.97 (1.45,2.68) | < 0.001 |
| Charlson comorbidity index | 1.14 (1.09,1.19) | < 0.001 |
| SOFA score | 1.07 (1.02,1.12) | 0.007 |
| SAPS Ⅱ | 1.05 (1.04,1.05) | < 0.001 |
| Antibiotic(day1) | 1.03 (0.58,1.86) | 0.91 |
| Vasoactive agent (day1) | 2.36 (1.69,3.30) | < 0.001 |
| Creatinine | 1.26 (1.16,1.36) | < 0.001 |
| BUN | 1.02 (1.01,1.02) | < 0.001 |
| Hematocrit | 0.99 (0.98,1.01) | 0.597 |
| Glucose | 1.00 (0.99,1.00) | 0.478 |
| Platelets) | 1.00 (0.99,1.00) | 0.553 |
| WBC | 0.99 (0.98,1.01) | 0.786 |
| Hemoglobin | 0.96 (0.91,1.02) | 0.156 |
| ALT | 1.00 (1.00,1.00) | 0.057 |
| RDW | 1.17 (1.13,1.23) | < 0.001 |

Note: HR, hazard ratio; CI, confidence interval; MBP, mean blood pressure; SOFA, Sequential Organ Failure Assessment; BUN, blood urea nitrogen; WBC, white blood cell; ALT, alanine aminotransferase.
