# Supplementary material for: Association between red cell distribution width and 30-day mortality in patients with sepsis-associated liver injury: a retrospective cohort study
Source: Front Med (Lausanne). 2024 Dec 18;11:1510997. doi: 10.3389/fmed.2024.1510997 (PMC11688371; doi:10.3389/fmed.2024.1510997)
Supplement: Supplementary file 5 [file Table_5.docx]

Supplementary Table 5 ROC analysis of RDW, SOFA, and SAPS II.

| variables | AUC | 95%CI | Cut-off value | Sensitivity | Specificity | Youden’s index |
| --- | --- | --- | --- | --- | --- | --- |
| RDW | 0.704 | 0.658-0.750 | 16.55 | 0.559 | 0.757 | 0.316 |
| SOFA | 0.566 | 0.515-0.617 | 5.50 | 0.452 | 0.669 | 0.121 |
| SAPS II | 0.800 | 0.761-0.838 | 49.50 | 0.777 | 0.704 | 0.481 |

Note: ROC, receiver operating characteristic; RDW, red blood cell distribution width; SOFA, Sequential Organ Failure Assessment; SAPS II, simplified acute physiology score; AUC, area under the curve; CI, confidence interval.
