# Supplementary material for: Association between red cell distribution width and 30-day mortality in patients with sepsis-associated liver injury: a retrospective cohort study
Source: Front Med (Lausanne). 2024 Dec 18;11:1510997. doi: 10.3389/fmed.2024.1510997 (PMC11688371; doi:10.3389/fmed.2024.1510997)
Supplement: Supplementary file 6 [file Table_6.docx]

Supplementary Table 6 Relationship between red blood cell distribution width and 30-day morality in different models.

| Item | n. total | n. event(%) | Model 1 | | Model 2 | | Model 3 | |
| --- | --- | --- | --- | --- | --- | --- | --- | --- |
|  |  |  | HR (95%CI) | p-value | HR (95%CI) | HR (95%CI) | HR (95%CI) | p-value |
| RDW | 529 | 188 (35.5) | 1.17 (1.13~1.23) | <0.001 | 1.18 (1.13~1.23) | <0.001 | 1.14 (1.09~1.19) | <0.001 |
| RDW-Group |  |  |  |  |  |  |  |  |
| ≤15.5 | 285 | 67 (23.5) | 1(Ref) |  | 1(Ref) |  | 1(Ref) |  |
| ＞15.5 | 244 | 121 (49.6) | 2.41 (1.79~3.25) | <0.001 | 2.35 (1.74~3.18) | <0.001 | 2.01 (1.48~2.72) | <0.001 |
| RDW, Quartiles |  |  |  |  |  |  |  |  |
| Q1 | 128 | 19(14.8) | 1(Ref) |  | 1(Ref) |  | 1(Ref) |  |
| Q2 | 126 | 34(27.0) | 1.95 (1.11~3.42) | 0.02 | 1.88 (1.07~3.30) | 0.028 | 1.53 (0.86~2.71) | 0.149 |
| Q3 | 140 | 55(39.3) | 2.98 (1.77~5.03) | <0.001 | 2.83 (1.68~4.78) | <0.001 | 2.05 (1.19~3.51) | 0.009 |
| Q4 | 135 | 80(59.3) | 5.21 (3.16~8.61) | <0.001 | 5.06 (3.07~8.36) | <0.001 | 4.50 (2.68~7.58) | <0.001 |
| Trend test |  |  |  | <0.001 |  | <0.001 |  | <0.001 |

Model 1: unadjusted

Model 2: adjust for age, race

Model 3: adjust for model 2+heart rate, MBP, creatinine, BUN, ALT, Charlson comorbidity index, SOFA, SAPS Ⅱ, malignant cancer, vasoactive agent (day1).

Note: BUN, creatinine, and ALT were categorized based on clinically relevant cutoff values. SOFA score and the Charlson Comorbidity Index were categorized using their respective medians. HR, hazard ratio; CI, confidence interval; RDW, red blood cell distribution width; MBP, mean blood pressure; SOFA, Sequential Organ Failure Assessment; SAPS II, simplified acute physiology score; BUN, blood urea nitrogen; ALT, alanine aminotransferase.
