# Supplementary material for: Association between red cell distribution width and 30-day mortality in patients with sepsis-associated liver injury: a retrospective cohort study
Source: Front Med (Lausanne). 2024 Dec 18;11:1510997. doi: 10.3389/fmed.2024.1510997 (PMC11688371; doi:10.3389/fmed.2024.1510997)
Supplement: Supplementary file 7 [file Table_7.docx]

Supplementary Table 7 Relationship between red blood cell distribution width and 30-day morality in different models after excluding patients with liver malignancies.

| Item | n. total | n. event(%) | Model 1 | | Model 2 | | Model 3 | |
| --- | --- | --- | --- | --- | --- | --- | --- | --- |
|  |  |  | HR (95%CI) | p-value | HR (95%CI) | p-value | HR (95%CI) | p-value |
| RDW | 480.0 | 156 (32.5) | 1.15 (1.1~1.21) | <0.001 | 1.16 (1.1~1.22) | <0.001 | 1.15 (1.08~1.22) | <0.001 |
| RDW-Group |  |  |  |  |  |  |  |  |
| ≤15.5 | 273.0 | 62 (22.7) | 1(Ref) |  | 1(Ref) |  | 1(Ref) |  |
| ＞15.5 | 207.0 | 94 (45.4) | 2.22 (1.61~3.06) | <0.001 | 2.17 (1.57~2.99) | <0.001 | 1.62 (1.17~2.23) | 0.004 |
| RDW, Quartiles |  |  |  |  |  |  |  |  |
| Q1 | 118.0 | 16 (13.6) | 1(Ref) |  | 1(Ref) |  | 1(Ref) |  |
| Q2 | 118.0 | 34 (28.8) | 2.31 (1.27~4.18) | 0.006 | 2.23 (1.23~4.04) | 0.008 | 1.68 (0.92~3.06) | 0.091 |
| Q3 | 120.0 | 41 (34.2) | 2.83 (1.59~5.04) | <0.001 | 2.72 (1.53~4.86) | 0.001 | 1.85 (1.02~3.35) | 0.044 |
| Q4 | 124.0 | 65 (52.4) | 4.65 (2.69~8.04) | <0.001 | 4.47 (2.58~7.72) | <0.001 | 2.92 (1.64~5.19) | <0.001 |
| Trend test |  |  |  | <0.001 |  | <0.001 |  | <0.001 |

Model 1: unadjusted

Model 2: adjust for age, race

Model 3: adjust for model 2+heart rate, MBP, creatinine, BUN, ALT, Charlson comorbidity index, SOFA, SAPS Ⅱ, malignant cancer, vasoactive agent (day1)

Note: HR, hazard ratio; CI, confidence interval; RDW, red blood cell distribution width; MBP, mean blood pressure; SOFA, Sequential Organ Failure Assessment; SAPS II, simplified acute physiology score; BUN, blood urea nitrogen; ALT, alanine aminotransferase.
