# Supplementary material for: Association between red cell distribution width and 30-day mortality in patients with sepsis-associated liver injury: a retrospective cohort study
Source: Front Med (Lausanne). 2024 Dec 18;11:1510997. doi: 10.3389/fmed.2024.1510997 (PMC11688371; doi:10.3389/fmed.2024.1510997)
Supplement: Supplementary file 8 [file Table_8.docx]

Supplementary Table 8 Relationship between RDW and 30-day morality after excluding patients with liver malignancies, anemia, and myelodysplastic syndromes.

| Item | n. total | n. event(%) | Model 1 | | Model 2 | | Model 3 | |
| --- | --- | --- | --- | --- | --- | --- | --- | --- |
|  |  |  | HR (95%CI) | p-value | HR (95%CI) | p-value | HR (95%CI) | p-value |
| RDW | 278.0 | 93 (33.5) | 1.19 (1.11~1.27) | <0.001 | 1.19 (1.11~1.28) | <0.001 | 1.24 (1.13~1.37) | <0.001 |
| RDW-Group |  |  |  |  |  |  |  |  |
| ≤15.5 | 173.0 | 40 (23.1) | 1(Ref) |  | 1(Ref) |  | 1(Ref) |  |
| ＞15.5 | 105.0 | 53 (50.5) | 2.49 (1.65~3.76) | <0.001 | 2.41 (1.59~3.63) | <0.001 | 1.69 (1.05~2.73) | 0.031 |
| RDW, Quartiles |  |  |  |  |  |  |  |  |
| Q1 | 69.0 | 11 (15.9) | 1(Ref) |  | 1(Ref) |  | 1(Ref) |  |
| Q2 | 66.0 | 16 (24.2) | 1.6 (0.74~3.46) | 0.228 | 1.57 (0.73~3.39) | 0.249 | 1.08 (0.49~2.41) | 0.842 |
| Q3 | 69.0 | 24 (34.8) | 2.53 (1.24~5.17) | 0.011 | 2.46 (1.2~5.04) | 0.014 | 1.7 (0.8~3.6) | 0.167 |
| Q4 | 74.0 | 42 (56.8) | 4.4 (2.26~8.55) | <0.001 | 4.13 (2.12~8.04) | <0.001 | 2.52 (1.22~5.21) | 0.013 |
| Trend test |  |  |  | <0.001 |  | <0.001 |  | 0.003 |

Model 1: unadjusted

Model 2: adjust for age, race

Model 3: adjust for model 2+heart rate, MBP, creatinine, BUN, ALT, Charlson comorbidity index, SOFA, SAPS Ⅱ, malignant cancer, vasoactive agent (day1)

Note: HR, hazard ratio; CI, confidence interval; RDW, red blood cell distribution width; MBP, mean blood pressure; SOFA, Sequential Organ Failure Assessment; SAPS II, simplified acute physiology score; BUN, blood urea nitrogen; ALT, alanine aminotransferase.
