# Supplementary figures and images for: Association between red cell distribution width and 30-day mortality in patients with sepsis-associated liver injury: a retrospective cohort study
Source: Front Med (Lausanne). 2024 Dec 18;11:1510997. doi: 10.3389/fmed.2024.1510997 (PMC11688371; doi:10.3389/fmed.2024.1510997)

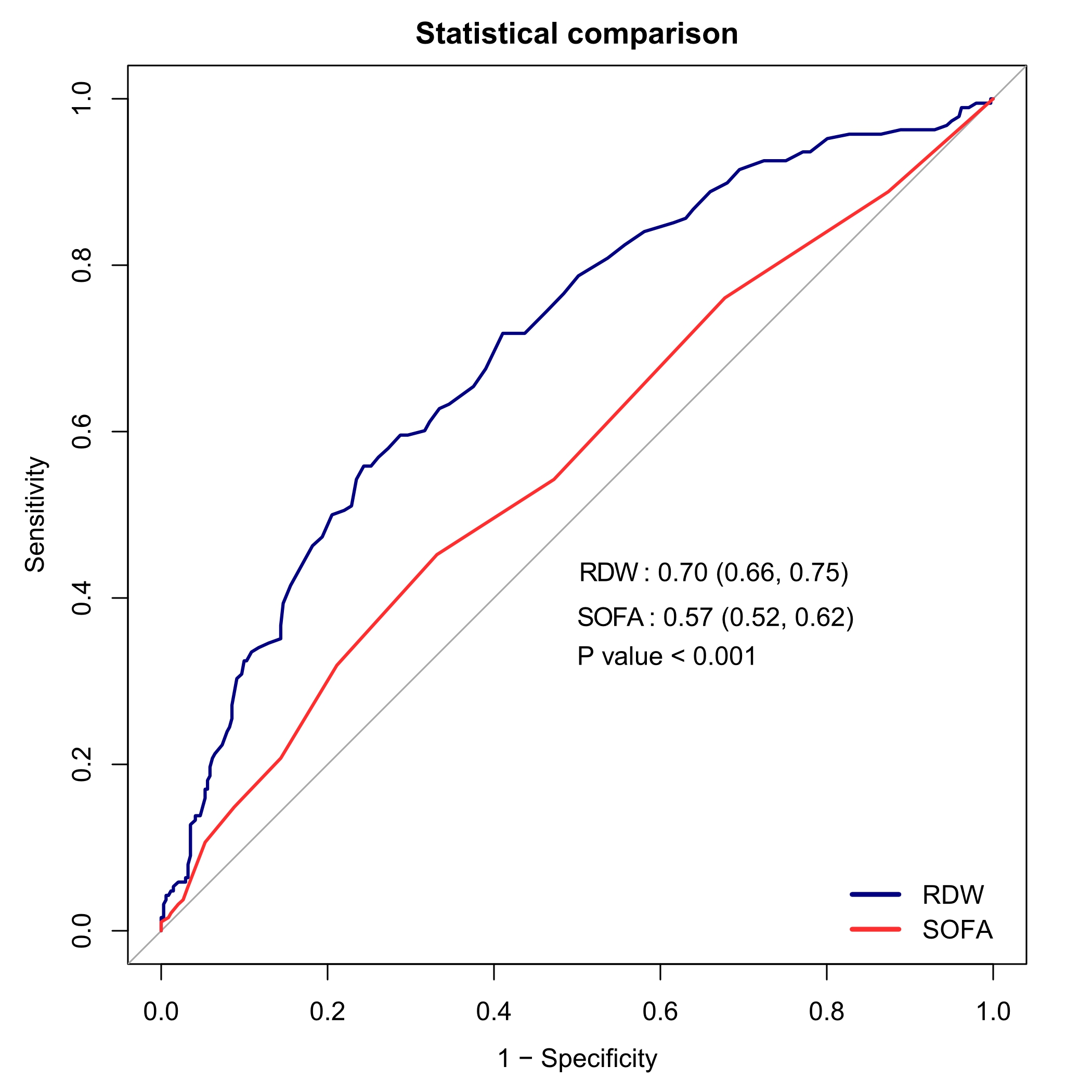

Supplement: Supplementary file 9 [file Image_1.jpeg]

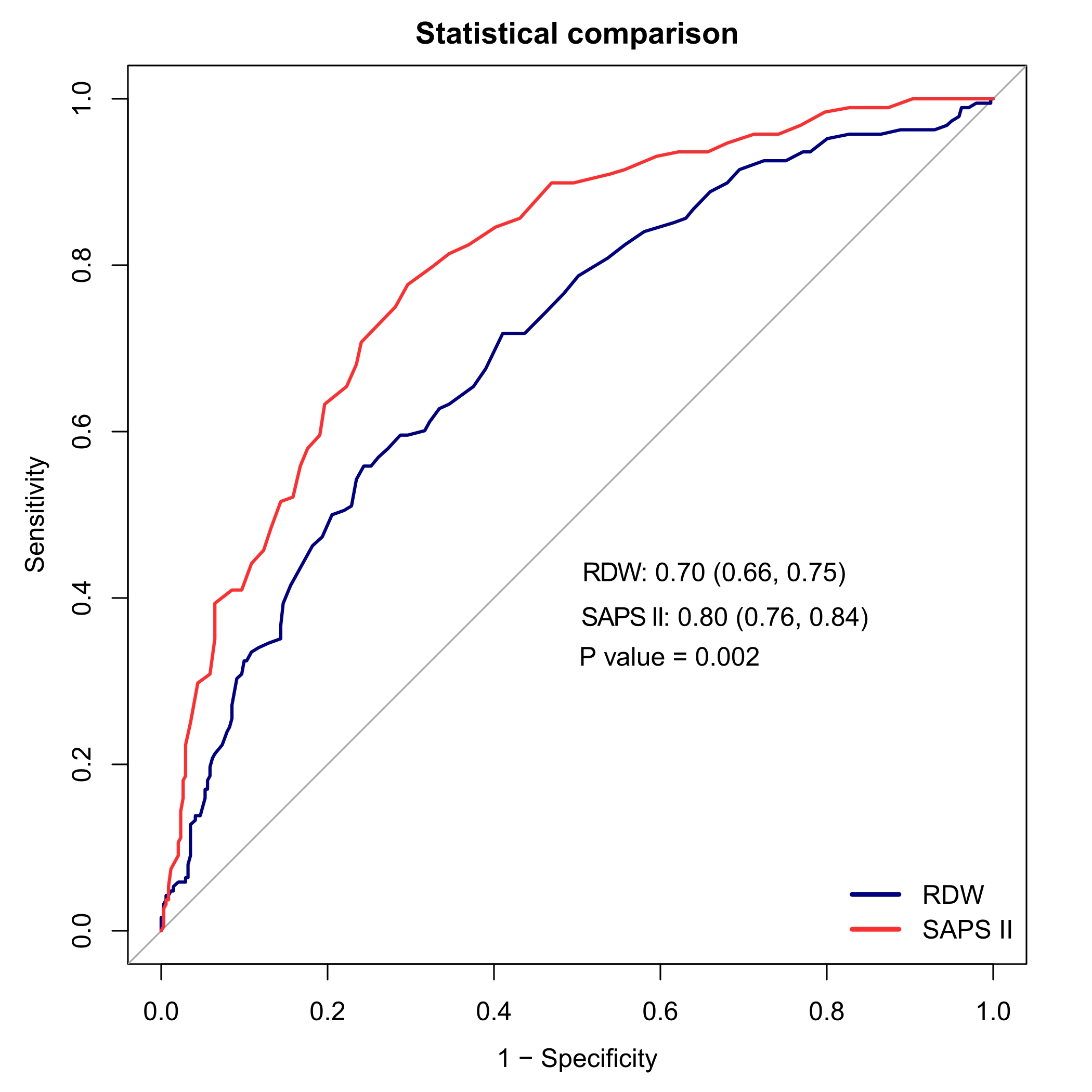

Supplement: Supplementary file 10 [file Image_2.jpeg]

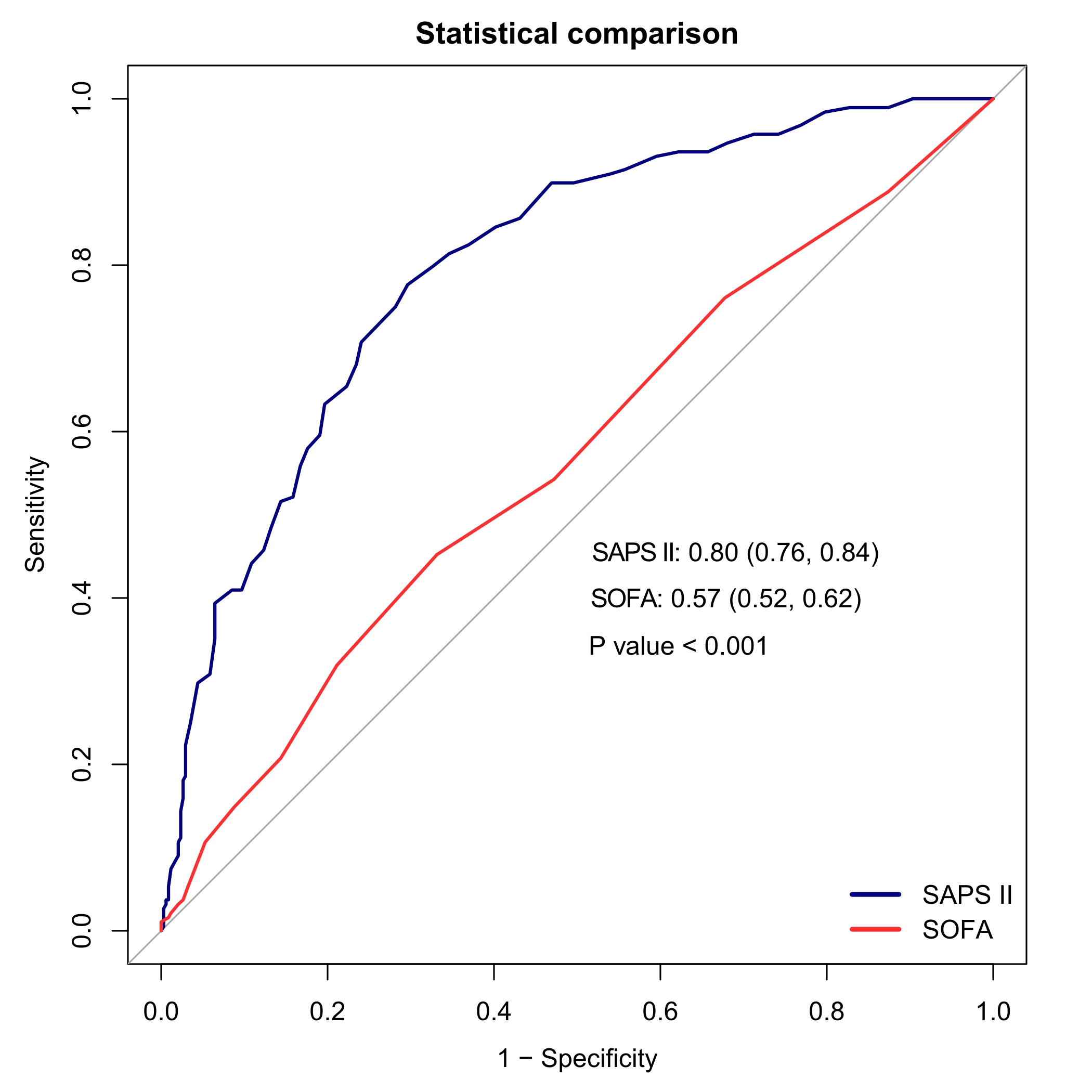

Supplement: Supplementary file 11 [file Image_3.jpeg]

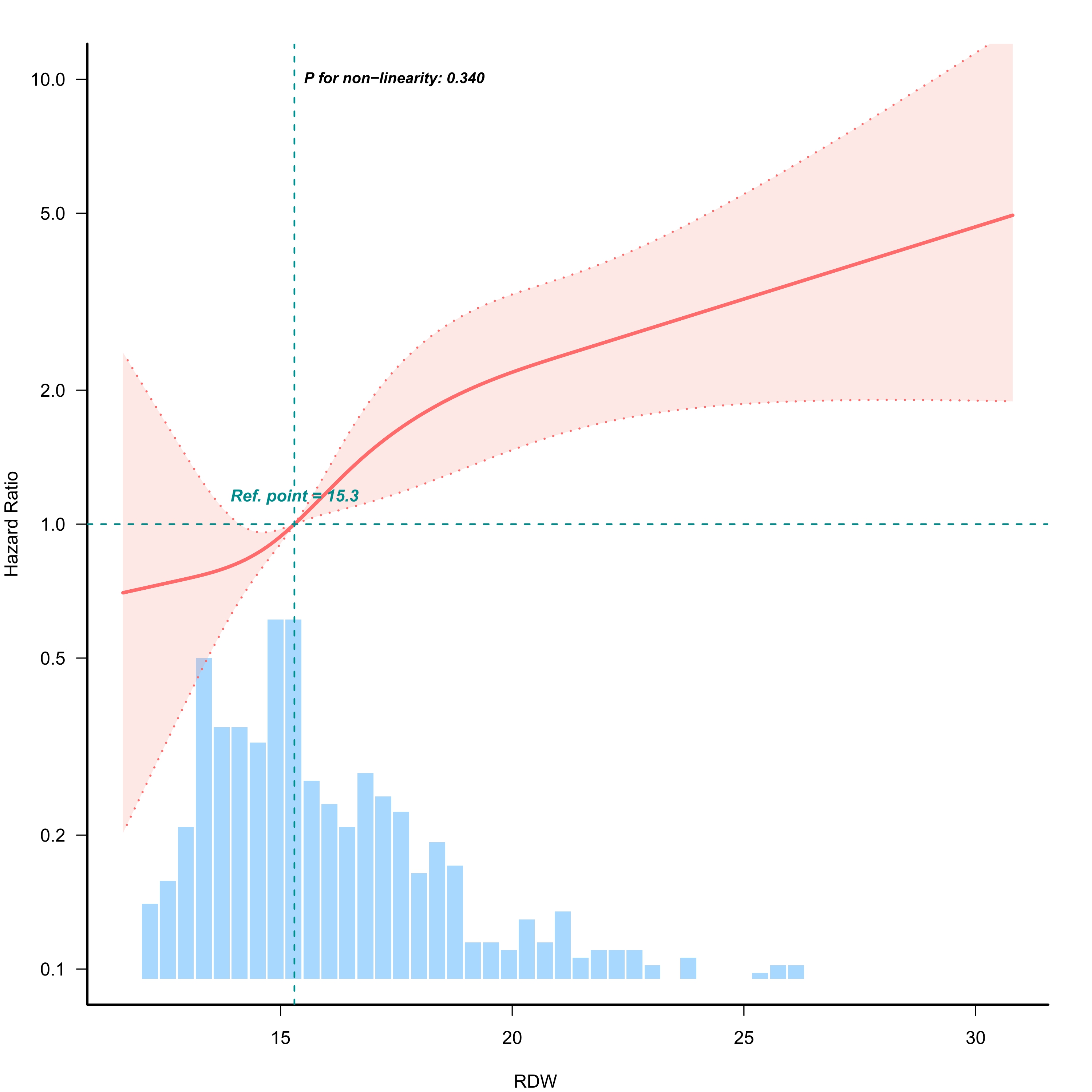

Supplement: Supplementary file 12 [file Image_4.jpeg]
